# Isolation and characterization of endothelial progenitor cells from Rhesus monkeys

**DOI:** 10.1186/2050-490X-2-5

**Published:** 2014-03-03

**Authors:** Wen Sun, Lily Zheng, Pengfei Han, Y James Kang

**Affiliations:** Regenerative Medicine Research Center, West China Hospital, Sichuan University, Chengdu, Sichuan, 610041 China

**Keywords:** EPCs, Isolation, Cell culture, Cell proliferation, Characterization, Monkey

## Abstract

**Background:**

Endothelial progenitor cells (EPCs) are increasingly becoming a major focus of regenerative medicine research and practice. The present study was undertaken to establish an appropriate procedure for isolation and characterization of EPCs from Rhesus monkeys for regenerative medicine research.

**Result:**

Selective CD34+ and nonselective mononuclear EPCs were isolated from bone marrow and cultured under varying conditions. The results showed that nonselective mononuclear EPCs were a better choice for high yield of the target cells. The cells grew in M 200 better than in EGM-2, and supplementation with fetal bovine serum promoted cell proliferation; but serum level at 7.5% was better than at 10%. In addition, surface coating of the culture dishes with human fibronectin significantly improved the proliferation and ontogeny of the isolated EPCs. Immunocytochemistry including detection of markers CD34, CD133 and CD31 and double-staining for Ac-LDL and lectin verified the purity of the cultured mononuclear EPCs.

**Conclusion:**

By a thorough analysis, we established a practical procedure for isolation and propagation of EPCs from Rhesus monkeys. This procedure would help using these valuable cells for regenerative medicine research.

## Background

Endothelial progenitor cells (EPCs) are increasingly becoming a major focus of regenerative medicine research and practice. These cells are enriched in the mononuclear cell fraction of peripheral blood but have also been isolated from bone marrow, the vessel wall, and a number of other organs and tissues [1–3]. Both experimental [4–7] and human clinical trials of EPC-based therapies [8–10] have generated encouraging results that underscored the significance of this cell type in cardiovascular medicine; a role for EPCs in the modulation of angiogenesis has been recognized [1–3]. However, there are a number of elusive issues in a comprehensive understanding of the usefulness of EPCs. For instance, it is unknown for the precise ontogeny and lineage of these cells, the true extent to which EPCs participate in neovascularization and vascular repair, and the efficacy of EPC-based regenerative therapies [11–13].

The encouraging data for the clinically potential of EPCs and the challenges for their development for clinical use have prompted an explosion of interest in experimental and clinical understanding of EPCs. Studies have focused on the role of EPCs in postnatal vasculogenesis in human health and disease [14–18]. In this context, purification and characterization of EPCs from rodent animal models have generated exciting data [16, 19–21]. However, there are significant limitations in these rodent studies, and the most noticeable restriction is the uncertainty of the extrapolation of the experimental data to humans due to remarkable variations in many aspects of physiology and pathogenesis between rodents and humans [22].

Non-human primate models of human disease and pathogenesis have become increasingly demanded experimental models for cardiovascular medicine. This is due largely to the emphasis of translational medicine research and the close similarity between non-human primates and humans in many aspects of cardiovascular physiology and pathology [23, 24]. With regard to the study of EPCs in cardiovascular medicine, the monkey model would provide an excellent assessment for the development of their potential for clinical application. It is thus of significant experimental and clinical interest to develop a procedure for the purification and characterization of EPCs isolated from monkeys for the purpose stated above.

In the present study, we carefully analyzed the effects of different procedures including purification, culturing conditions, culture medium compositions, and selection of culture glassware on EPCs isolated from Rhesus monkeys. An optimized procedure was developed to achieve a high yield and stable culture of the EPCs from monkeys. This procedure would greatly enhance the efficacy and reliability of the cultured EPCs isolated from monkeys for the studies of their growth and differentiation, as well as their lineage and participation in neovascularization.

## Results

### Effects of isolation procedures on the yield and characterization of EPCs isolated from Rhesus monkeys

**Figure 1:**
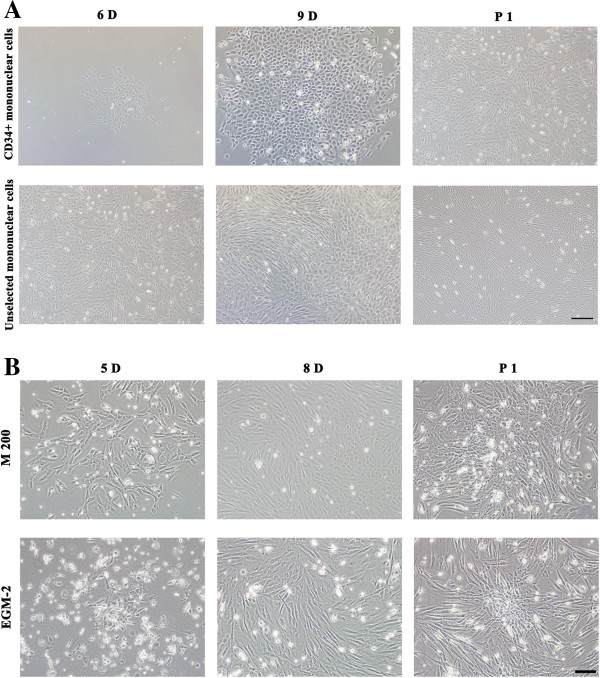
**The morphology of EPCs isolated from Rhesus monkeys. A**. Selected CD34+ mononuclear cells and unselected mononuclear cells were cultured in M 200, observed on day 6 (6D) or day 9 (9D) after culturing and day 4 after the first passage (P1). Both selected CD34+ and unselected mononuclear cells were cerioid shape on day 6, formed cobblestone-appearance colonies on day 9, and become more spindle shape with the loss of stereognosis after first passage. Bar =200 μm. **B**. Unselected mononuclear cells were cultured in either M 200 or EGM-2 media, observed on day 5 (5D), day 8 (8D) after culturing and day 1 after the first passage (P1). Cells cultured in M 200 proliferated faster than in EGM-2, but cells in EGM-2 had better stereognosis. Bar =100 μm.

Mononuclear EPCs isolated from bone marrow by gradient centrifugation were either directly cultured or subjected to CD34-affinity column for further purification. The data presented in Figure 1A show the difference in the morphology of the EPCs in cultures. Adherent CD34+ mononuclear cells grew into cerioid colonies after 5-7 days; cobblestone-appearance colonies were observed after 9-11 days; and spindle shaped colonies existed all the time. On the 4th day after the first passage, which was conducted approximately 2 weeks after the palting, cells grew more uniformly with the increased spindle shaped cells and losing of stereognosis.

Adherent unselected mononuclear EPCs grew into cerioid colonies after 4-6 days, and spindle shaped and cobblestone-appearance colonies were both observed during further culturing. The first passage was conducted 8-10 days after the plating and cell morphology changed with the increased spindle shaped cells and losing of stereognosis observed on the 1^st^ day after 1^st^ passage.

Comparing these two isolation methods, selecting CD34+ mononuclear cells required more time, but the yield of CD34+ mononuclear cells was lower than unselected cells, and the CD34+ cells grew much slower. Therefore, unselected mononuclear EPCs were chosen to perform the following experiments.

### Effects of culture media on EPCs

The unselected mononuclear EPCs were equally divided and cultured in two different media: M 200 and EGM-2. Figure 1B shows the difference in morphology of the EPCs under two conditions. Both spindle shaped and cobblestone-appearance cells were observed in M 200 and EGM-2. Cells cultured in M 200 proliferated faster than in EGM-2, but cells had better stereognosis in EGM-2.

### Effects of serum concentrations on EPCs

**Figure 2:**
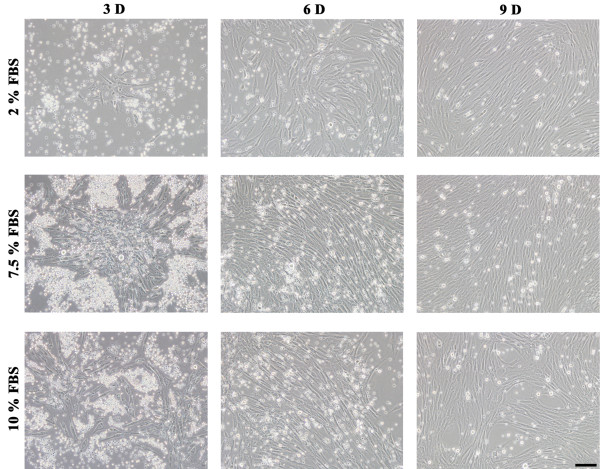
**Effects of serum concentrations on EPCs in cultures. Unselected mononuclear cells were cultured in M 200 or M 200 supplemented with 7.5%** **or 10%** **FBS,** **observed on day 3,** **day 6 and day 9.** Cells in the medium supplemented with FBS grew faster than in medium without FBS supplementation, but cells in the medium supplemented with 7.5% FBS proliferated better than in the medium supplemented with 10% FBS. Bar =100 μm.

The unselected mononuclear EPCs were cultured in media supplemented with varying concentrations of FBS, as shown in Figure 2. On the third day, cells in the media supplemented with FBS grew faster than in media without FBS supplementation, and this phenomenon was more obvious on the 6th day and last until the 9th day. However, cells cultured in media containing 7.5% FBS proliferated better than in media supplemented with 10% FBS.

### Effects of culture dishes surface coating on EPCs

**Figure 3:**
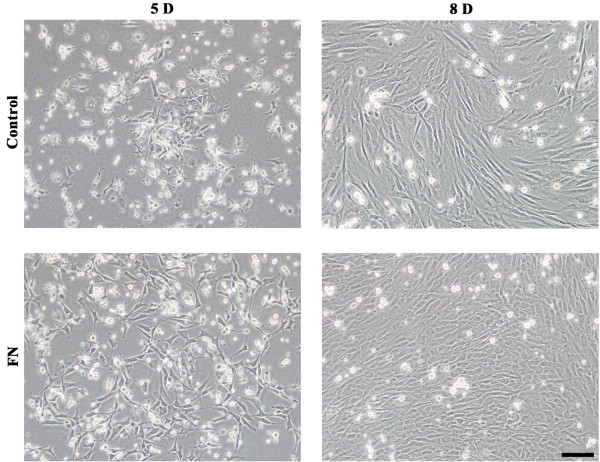
**Effects of culture dish surface coating on EPCs in cultures.** Unselected mononuclear cells were seeded in EGM-2 medium without serum supplementation on FN coated culture dishes. Morphology of EPCs was respectively observed on day 5 and day 8 after culturing. In the control group, cells were seeded on culture dishes without coating. Cells seeded on FN coated dishes had better adherence than cells seeded on dishes without coating 5 days after culturing, which remained observable until 8 days after culturing. Bar =100 μm.

The unselected mononuclear EPCs were plated on culture dishes without coating or coated with 25 μg/ml FN, as shown in Figure 3. On the 5th day, cells seeded on FN-coated dishes adhered much better than cells cultured in dishes without coating, which remained observable until on the 8th day. Comparing the effects between FBS supplementation and FN coating, we found that the effect of FN coating was similar with FBS supplementation, but FBS jeopardizes the multipotential of stem cells [25].

### Characterization of cells in cultures

**Figure 4:**
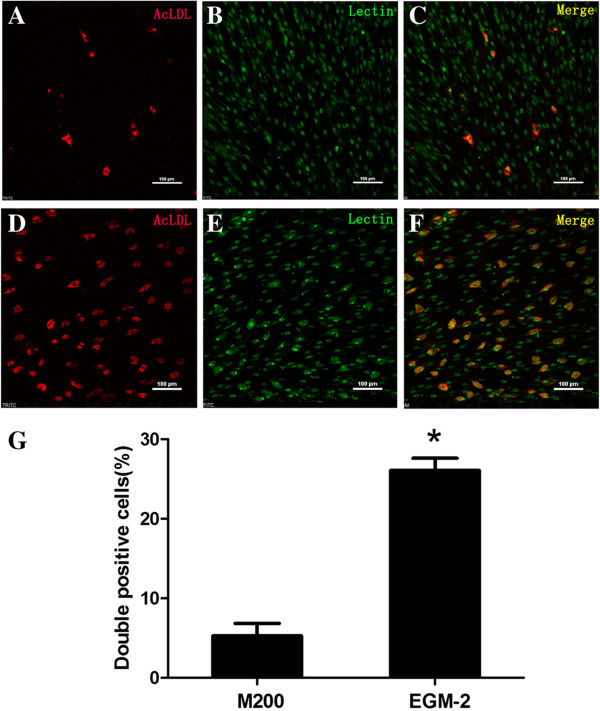
**Characterization of cells in cultures by staining cells with FITC**-**lectin and examing uptake of Dil AcLDL.** Unselected mononuclear EPCs were cultured on FN coated dishes and in M 200 or EGM-2 medium, and characterization was proceeded by uptake of Dil AcLDL (left, red) and FITC-lectin staining (middle, green). Double positive cells (right, merge) were observed in both M 200 **(A-C)** and EGM-2 **(D-F)** on the 19^th^ day after plating, but the number of double positive cells in EGM-2 was much more than in M 200 **(G)**. *, significantly different (P <0.05). Bar =100 μm.

Unselected mononuclear EPCs were cultured on human FN coated culture dishes and in M 200 or EGM-2 without FBS supplementation. Under this condition, cells were characterized by surface markers CD34, CD133, CD31, by FITC-lectin staining, and by the uptake of Dil AcLDL. FITC-lectin staining and Dil AcLDL uptake are classical methods for EPCs characterization; binding of lectin is specific for human endothelial cells, and uptake of Dil AcLDL is a function associated with endothelial cells [3, 26, 27]. There were no differences in the expression of CD34, CD133, and CD31 between the two different media (data not shown). The data presented in Figure 4 show that almost all the cells were bound with FITC-lectin, but uptake of Dil AcLDL was only observed in a portion of cell population. Importantly, there was much more double-positive staining of FITC-lectin and Dil AcLDL in cells cultured in EGM-2 than those in M 200.

## Discussion

There are several reports on isolation and culturing of EPCs from rodent models [16, 28]. However, this is the first report to describe a procedure to isolate and culture EPCs from Rhesus monkeys. There were no special requirements for the procedure, but there were specific details to which attention needed to be paid. Separation of CD34+ mononuclear cells would be considered an ideal procedure to purify EPCs, but the low yield in addition to time-consuming would make it less practical for a large scale collection of these cells. Selection of different types of media made a remarkable difference in the maintenance of morphology of the isolated EPC. It was also found that serum supplementation was required, but high concentrations of sera resulted in more differentiation of EPCs in cultures. Finally, a better result was obtained if the culture dishes were coated with FN relative to uncoated culture ware.

There were reports on isolating EPCs using selected CD34+ or unselected procedures. It appears that the procedure using selected CD34+ mononuclear cells would generate relatively purified EPCs. However, studies have also shown that there was no remarkable advance using the selected CD34+ cells over the unselected mononuclear cells in cultures [26, 27, 29]. In the present study, both procedures were conducted to make a close comparison. The results obtained show that there were no apparent differences in morphology of EPCs in cultures, but there were significant differences in time-consuming and yield between the two procedures. The lower yield of CD34+ mononuclear cells might not reflect an insufficient number of CD34+ cells in the population, but rather resulted from less efficient procedure to separate CD34+ cells using the CD34+ MicroBeads. Considering all of these drawbacks in the current procedure separating CD34+ cells, we recommend that directly using unselected mononuclear cells for the study of EPCs isolated from monkey models.

The differences between M 200 and EGM-2 media include compositions and quantity of the same composition. Although remarkable differences in cell morphology in cultures were not observed between cells cultured in the two differenct media, cells cultured in M 200 proliferated faster than in EGM-2, and cells had better stereognosis in EGM-2. These alterations would suggest that a selection of media for culturing EPCs should consider the need for the eventual application. However, the EGM-2 would a better medium to maintain undifferentiated shape for a longer time of culturing.

It is well known that serum is essential for cell growth and high levels of sera stimulate cell proliferation. It is difficult to define the optimal concentrations of sera in cultures for maintaining undifferentiated morphology of EPCs in cultures but sufficiently supporting cell growth. In this study, we found that serum concentrations at 7.5% in media would be a better supplementation, and at 10% promoted differentiation. Therefore, this would provide a reference in the study of EPCs isolated from monkeys.

FN coated culture dishes provide better adherent support for cells in cultures. In the present study, it appeared that FN coating not only provided an adherent support for EPCs, but also helped cells maintain undifferentiated morphorlogy and promote proliferation. The cell growth promotion observed in the FN coated dishes was comparable with that observed in the cultures supplemented with 7.5% FBS. This procedure is thus strongly recommended for culturing EPCs isolated from monkeys.

## Conclusions

Above all, an optimized procedure was developed: unselected mononuclear cells were cultured on human FN coating culture dishes and in EGM-2 without additional FBS supplementation. Under this condition, spindle shaped and cobblestone-appearance cells displayed a better adherence and growth, and were identified to be EPCs.

## Methods

### Animals

Male Rhesus (Macaca mulatta) monkeys, aged 2-3 years old and weighed 4.5 to 6.0 kg, were obtained from Chengdu Ping-An experimental animal breeding and research center, a Chinese government accredited non-human primate center in Sichuan province. The animals were housed in individual cages and acclimatized to the laboratory condition for a period of at least one month. They received conventional laboratory diet with free access to drinking water, which was approved by the Laboratory Animal Management Committee of Sichuan province. All animal procedures were approved by the Institutional Animal Care and Use Committee at the Sichuan University West China Hospital, following the guideline of the US National Institutes of Health.

### Isolation of mononuclear cells

Prior to experimental procedure, all subjects received an intramuscular injection of 5 mg/kg ketamine and 0.2 mg/kg midazolam to induce sedation. The hairs covering the iliums or knees at the puncturing sites were shaved and the exposed skin was sterilized by iodophor and alcohol in sequence. Bone marrow puncture was made by using puncture needles and approximately 4-6 ml bone marrow was aspirated from one or both iliums (or femurs) under strict sterile condition.

The aspirate was diluted with 4-5 ml L-DMEM media (Gibco, USA), mixed carefully and added on the top of 5-6 ml lymphocyte separation media (density 1.077 g/ml, TBD, China) at room temperature. After gradient centrifugation at 600 × g for 20 min, the yellow-white plate (mononuclear cells) was collected, washed and cultivated in media directly or further undergone separation of CD34+ cells.

### Separation of CD34+ cells

CD34+ cells were separated using human CD34 MicroBead Kit (Miltenyi Biotec, Germany) following the instruction provided. In brief, mononuclear cell suspension was centrifuged at 300 × g for 10 min. The cell pellet was resuspended in 300 μL of autoMACS™ running buffer (Miltenyi Biotec, Germany), 100 μL of FcR blocking reagent, and 100 μL of CD34 MicroBeads. After incubation for 30 min in the refrigerator (2 − 4°C), the cells attached to the CD34 MicroBeads were washed with 5 − 10 mL of autoMACS™ running buffer followed by centrifugation at 300 × g for 10 min. The cell pellet was resuspended in 500 μL of autoMACS™ running buffer, and applied onto an autoMACS™ running buffer rinsed column on a magnetic separator. The colume was washed 3 times and removed from the separator. The cells were flushed out from the colume using 1.0 mL of autoMACS™ running buffer. The collected cells were washed and cultivated.

### Cell culture

Selected CD34+ and unselected mononuclear cells were cultured in media supplemented with low serum growth supplement (LSGS, Gibco, USA) containing fetal bovine serum (FBS), hydrocortisone, human epidermal growth factor (hEGF), basic fibroblast growth factor (bFGF), heparin, and antibiotics (penicillin and streptomycin, Thermo, USA).

#### Effects of culture media

Cells were equally divided and cultured in two different media. One portion was cultured in Medium 200 (M 200) supplemented with low serum growth supplement. Another portion was cultured in EGM-2 medium, which is the endothelial cell basal medium-2 plus EGM-2 MV SigleQuots (Lonza, USA), a supplement kit containing FBS, hydrocortisone, hFGF-B, VEGF, R3-IGF-1, ascorbic acid, hEGF, GA-1000, and antibiotics (penicillin and streptomycin, Thermo, USA).

#### Effects of serum concentrations

Media were supplemented with FBS at different concentrations: medium with its own serum (serum concentration in M 200 was 2%, and in EGM-2 was 5%), and media with added FBS to the final concentration of 7.5% or 10% in the medium.

#### Effects of culture dish surface coating

Cells were plated on uncoated culture dishes (BD, USA) or human fibronectin (FN, Prospec, Israel) coated culture dishes. The coating was done as follow: 1 ml of 25 μg/ml human FN was added to the dishes and incubated at room temperature for 2 hrs, after which the supernatant was removed and dishes were rinsed with PBS buffer.

The first change of media was conducted 3-5 days after the plating. Afterwards, the media were changed every 3 days, with which non-adherent cells were washed away. Cells were digested and passaged at 80%-90% confluence for continuously culturing or characterization.

### Characterization of EPCs

The surface marker CD34 (Abcam, UK), CD133 (HuaAn, China) and CD31 (Abcam, UK) were detected by immuncytochemistry. In addition, cells were stained with FITC-labeled lectin (FITC-lectin, Sigma-Aldrich, USA). The uptake of Dil-conjugated acetylated low-density lipoprotein (Dil AcLDL, Invitrogen, USA) was also measured at the same time to define functional EPCs.

#### Immunocytochemistry

Cells were digested and seeded on sterile slides, which were put in 6-well plates. Before the detection, media were aspirated and PBS was added to wash cells three times, 5 min each wash. After fixed in cold 4% paraformaldehyde for 10-15 min and washed with PBS, primary antibodies diluted with PBS containing 10% serum was added and incubated for 2 hrs at 37°C followed by overnight at 4°C. The secondary antibody diluted with 10% serum in PBS was incubated for 1.5 hrs at 37°C. Then DAPI was incubated for 5-10 min at room temperature and slides were sealed after wash with PBS and examined with laser scanning confocal microscope (LSCM).

#### FITC-lectin staining and uptake of Dil AcLDL

Cells were seeded on 24-well plates, and undergone starvation for 24-48 hrs in media without serum, incubated with 200 μL 2.4 μg/ml Dil AcLDL 4 hrs at 37°C after medium removal and wash, and fixed in cold 4% paraformaldehyde for 10-15 min, followed by incubation in 200 μL 10 μg/ml FITC-lectin for 2 hrs at 37°C, and examined with LSCM after PBS washing.

### Statistical analysis

Data were obtained from three separate experiments and expressed as means ± S.E.M. The significance of difference between groups was determined by Student T-test. The level of significance was considered when P < 0.05.
